# *MDM4* contributes to the increased risk of glioma susceptibility in Han Chinese population

**DOI:** 10.1038/s41598-018-29468-6

**Published:** 2018-07-23

**Authors:** Peng Sun, Feng Yan, Wei Fang, Junjie Zhao, Hu Chen, Xudong Ma, Jinning Song

**Affiliations:** 1 Department of Neurosurgery, the First Affiliated Hospital of Xi’an Jiaotong University, Xi’an, Shaanxi China; 2 Department of Clinical Nutrition, Xijing Hospital, the Fourth Military Medical University, Xi’an, Shaanxi China; 3 Department of Neurosurgery, Tangdu Hospital, the Fourth Military Medical University, Xi’an, Shaanxi China

## Abstract

Recently, *MDM4* gene has been reported to be a susceptibility gene for glioma in Europeans, but the molecular mechanism of glioma pathogenesis remains unknown. The aim of this study was to investigate whether common variants of *MDM4* contribute to the risk of glioma in Han Chinese individuals. A total of 24 single-nucleotide polymorphisms (SNPs) of the *MDM4* gene were assessed in a dataset of 562 glioma patients (non-glioblastoma) and 1,192 cancer-free controls. The SNP rs4252707 was found to be strongly associated with the risk of non-GBM (*P* = 0.000101, adjusted odds ratio (OR) = 1.34, 95% confidence interval (CI) = 1.16–1.55). Further analyses indicated that there was a significant association between A allele of rs4252707 associated with the increased non-GBM risk. Haplotype analysis also confirmed a result similar to that of the single-SNP analysis. Using stratification analyses, we found the association of rs4252707 with an increased non-GBM risk in adults (≥18 years, *P* = 0.0016) and individuals without IR exposure history (*P* = 0.0013). Our results provide strong evidence that the *MDM4* gene is tightly linked to genetic susceptibility for non-GBM risk in Han Chinese population, indicating a important role for *MDM4* gene in the etiology of glioma.

## Introduction

Glioma is the most common primary central nervous system (CNS) tumor worldwide and accounts for approximately 80% of all brain tumors^1^. According to the 2007 World Health Organization (WHO) Classification of Tumors of the Central Nervous System, gliomas can be broadly classified into glioblastoma (GBM) and lower-grade non-GBM tumors. Recently, the revised 2016 WHO classification has included the tumor molecular features of IDH1/ATRX mutation and 1 p/19q co-deletion^2^. Although the annual incidence of glioma is 5.26 per 100,000 people, or 17,000 new diagnoses per year^3^, the etiology of glioma is very poorly understood. To date, high-dosage ionizing radiation (IR)^4^ and genetic variations^5^ have been identified as major risk factors for glioma. Exposure to moderate-to-high doses of IR typically causes DNA damage^6^, which increases the risk of glioma. An increased risk for first-degree relatives of patients with glioma has been observed, indicating that glioma may be partially explained by genetic factors^7^. In addition, only a small proportion of individuals who are exposed to IR environments will develop glioma, suggesting hereditary factors also contribute to susceptibility to glioma^8^. Hence, it is necessary to identify genes through candidate gene studies that are responsible for glioma susceptibility.

The mouse double minute 4 homolog gene (*MDM4*), also known as *MDMX*, is one of the members of the MDM family, which is composed of *MDM2*, *MDM4* and their derivatives^9^. One of the fundamental roles for these two proteins is the precise regulation of the tumor suppressor p53, which is vital for coordinated suppression of malignancy and cell survival^10^. Many studies have found that MDM4 was overexpressed in multiple primary human tumors, including breast cancer, lung cancer, colon cancer and retinoblastoma^11–13^. Approximately 40% of gliomas exhibit a p53 mutation or deletion, and approximately 80% of gliomas have a p53 pathway defect^14^, which may be caused by MDM4 overexpression. Previous studies showed that transcript variants of *MDM4* with a short internal deletion are more efficiently translocated to the nucleus than full-length MDM4, which generates much better suppressions of p53-mediated transcription, such as MDM4-B^15^ and MDM4-S^16^. The expression levels of these transcript variants were significantly associated with tumor stage^17^. Hence, *MDM4* may be involved in the onset and development of glioma. However, most studies focus on transcriptional levels, and little research has been designed to investigate the association between glioma and mutations of MDM4, which may be the root cause of the transcript variants of *MDM4*.

Understanding the genetic basis of complex human diseases has been increasingly emphasized. Recently, a meta-analysis of existing genome-wide association studies (GWASs) and two new GWASs of over 12,496 cases and 18,190 controls of European ancestry produced highly significant evidence of a strong association between the single-nucleotide polymorphism (SNP) rs4252707 (G/A) and non-glioblastoma glioma (*p*-value = 3.34 × 10^−9^, OR = 1.19)^18^. This SNP is located within intron 8 of the gene encoding MDM4. More importantly, the SNP rs4252707 shows strong linkage disequilibrium (LD) with rs12031912 and rs12028476, both of which map to the *MDM4* promoter^18^. These findings may suggest that *MDM4* is involved in the pathogenesis of glioma. However, these results were only identified in samples from patients of European ancestry. Given that different ethnic populations may exhibit glioma genetic heterogeneity, replications of the study using more samples from different populations are needed to confirm these results, and thus far, no information is available from the Han Chinese population regarding *MDM4*. Therefore, in the present study, we aimed to examine whether *MDM4* was associated with the risk of non-GBM in Han Chinese population.

## Materials and Methods

### Study subjects

In the present study, 562 patients with non-GBM glioma (351 men and 211 women) and 1,192 cancer-free individuals (746 men and 446 women) were enrolled from Tangdu Hospital at the Fourth Military Medical University between August 2013 and October 2016. To restrict the genetic background of our study subjects, all included patients were unrelated individuals from Shaanxi Province, and their immediate family members from the previous three generations were also born locally. All patients with non-GBM gliomas (astrocytomas, ependymomas, oligodendrogliomas and mixed glioma) were histopathologically diagnosed and confirmed by at least 2 pathologists. Tumor type and stage were classified according to the 2007 WHO classification^19^. The exclusion strategies for the enrolled patients were as follows: (1) patients with any history of cancer; (2) patients with previous and undergoing chemotherapy and radiotherapy. As controls, the cancer-free individuals were recruited based on the selection criteria of frequency-matched gender and age (±5 years) of the patients. Inclusion criteria of the control group were as follows: (1) individuals without central nervous system-related diseases, (2) individuals without recent infections, (3) individuals without any history of cancer, and (4) individuals without the history of chemotherapy or radiotherapy. The clinical and demographic information of all subjects are summarized in Table 1, including age, gender, IR exposure history, smoking status, family history of cancer and tumor type. Informed consent were signed by all subjects. This study strictly complied with the ethical guidelines of the Helsinki Declaration (2002 version) during the implementation process and was approved by the Medical Ethics Committee of the Fourth Military Medical University.

**Table 1:** The clinical and demographic information of the patient and control groups.

Characteristics	Subjects (N = 1,754)		*P*-value
	Patients	Controls	
Number	562	1,192	—
Age, mean ± SD (years)	39.95 ± 15.71	40.06 ± 15.94	0.895
Children (<18)	83	178	0.928
Adults (≥18)	479	1,014	
Gender			
Male	351	746	0.959
Female	211	446	
Ionizing radiation exposure history			
No	537	1,166	0.839
Yes	25	26	
Smoking status			
Non-smokers	296	629	0.969
Smokers	266	563	
Family history of cancer			
No	473	1,007	0.865
Yes	89	185	
Histology of non-GBM			
Astrocytomas	290	—	—
Oligodendrogliomas	119	—	—
Enpendymomas	102	—	—
Mixed glioma	51	—	—
WHO tumor grade			
I	98	—	—
II	224	—	—
III	240	—	—

### SNP selection and genotyping

We searched for all SNPs with minor allele frequencies (MAFs) ≥ 0.02 within the region of the *MDM4* gene in the 1000 Genomes Project Chinese Han Beijing population database. MAF ≥ 0.02 and tagging r^2^ ≥ 0.8 were used as a screening standard in the selection of tag SNP, which generated 24 tag SNPs for our study. As a result, these 24 tag SNPs (rs3014610, rs2169137, rs117139931, rs137991330, rs4252707, rs190876924, rs12024619, rs72644182, rs117137314, rs76605997, rs76432362, rs116854458, rs12138846, rs61421373, rs191840558, rs116907825, rs115517182, rs12567161, rs150337092, rs80242302, rs3789044, rs3789043, rs884108 and rs61817485) were included in further analyses. All our selected SNPs had *P* values greater than 0.05 by the HWE test. Commercial kits were used to extract genomic DNA from peripheral blood leukocytes (Genomic DNA kit, Axygen Scientific Inc., CA, USA). Genotyping was conducted for 24 selected SNPs by using the platform of Sequenom Mass ARRAY RS1000 system (Sequenom, San Diego, CA, USA). Typer Analyzer software (Sequenom, San Diego, California, USA) was used to process signal results to ultimately generate genotype data^20^. Case and control statuses were blinded during all genotyping processes for quality control. Five percent of the random samples were repeated, and the results were 100% concordant.

### Statistical analyses

We examined the differences in characteristic information between case and control groups. χ^2^ tests and Student’s t-tests were performed for categorical variables and continuous variables, respectively. HWE for all SNPs were calculated by Haploview v4.2. Single marker-based association analyses were conducted using Plink v1.9. Logistic models were fitted for each SNP, and age and gender were included as covariates to eliminate potential confounding effects. ORs and 95% confidence intervals (CIs) were reported. Bonferroni correction was conducted to balance multi-test correction. False-positive report probability (FPRP) was calculated to evaluate the significant results in the stratification analyses by using “R” software. A FPRP threshold of 0.2 with a prior probability of 0.1 were applied for examining the correlated risk, and FPRP values less than 0.2 were suggested to be noteworthy^21^. LD blocks were constructed using Haploview v4.2, and the haplotypic frequencies were calculated by GENECOUNTING v2.2. The differences in haplotypic frequencies between cases and controls were investigated, and haplotypic association analyses were performed. All statistical tests in the study were two-tailed, and p values less than 0.05 were considered statistically significant.

## Results

The clinical and demographic information of the patient and control groups are summarized in Table 1. Among the 562 non-GBM glioma cases, 290 patients had astrocytomas that were not classified as glioblastoma, and 272 had other types of gliomas, including ependymomas, oligodendrogliomas and mixed glioma. As shown in Table 1, we found no significant difference in age, smoking status, gender or ionizing radiation exposure history between case and control groups (*P* = 0.895, 0.959, 0.969 and 0.839, respectively). Approximately 16% of patients had family history of cancer, which was similar with that of the control groups (*P* = 0.865).

In the present study, a total of 24 SNPs within the *MDM4* gene were successfully genotyped in the samples of 562 cases and 1,192 controls, and all information is presented in Supplementary Table S1. The results of single SNP association analysis of 24 SNPs as well as Hardy-Weinberg equilibrium tests are shown in Table 2 and Supplementary Table S2. All SNP genotypes were in HWE (*P* > 0.05) (Table 2 and Supplementary Table S2). As shown in Table 2, we identified association signals for the SNP rs4252707 with non-GBM glioma risk by multivariate logistic regression analysis. The allelic *P* value was 0.000101 after accounting for the effects of age and gender. The significance remained (*P* = 0.002424) after Bonferroni correction (*P* × 24). Further analyses also suggested that A allele of rs4252707 was strongly correlated with the increased non-GBM risk (odds ratio (OR) = 1.34, with the adjustment of age and gender). Genotypic association analyses also confirmed a similar pattern of results (Table 2). An increased risk was associated with A carrier genotypes of rs4252707 compared with the non-A carrier genotype (Table 2). The other 23 SNPs did not differ significantly in their allelic or genotype distributions (Supplementary Table S2). Moreover, stratified analyses were conducted based on age, IR exposure history, smoking status and tumor type for the significant SNP (rs4252707). By stratification analyses, we found the association of rs4252707 with an increased non-GBM risk in adults (≥18 years, *P* = 0.0016), individuals without IR exposure history (*P* = 0.0013), all smokers and non-smokers (*P* < 0.05), and all patients with non-GBM glioma (*P* < 0.05) (Table 3). The results of FPRP analyses for significant findings in the subgroups were presented in Table 4. We found that all significant signals for the rs4252707 were still almost noteworthy at the prior probability level of 0.1 in the subgroups except for the subgroup of ependymomas. A possible reason was the smaller sample size than other subgroups, which resulted into an insufficient power.

**Table 2:** Allele and genotype frequency of single SNP association analyses.

SNP Affection	H-WE *P*-value	Allelic count (Freq. %)		Allelic *P*-value*	OR 95% CI^*^	Genotype count (Freq. %)			Genotypic *P*-value*
rs117139931		T	*C*			TT	TC	CC	
Case	0.522	1031 (91.73)	93 (8.27)	0.599012	1.01	474 (84.34)	83 (14.77)	5 (0.89)	0.663368
Control	0.461	2199 (92.24)	185 (7.76)		0.83–1.39	1016 (85.23)	167 (14.01)	9 (0.76)	
rs137991330		G	*A*			GG	GA	AA	
Case	0.591	1029 (91.55)	95 (8.45)	0.402046	1.12	472 (83.99)	85 (15.12)	5 (0.89)	0.386211
Control	0.210	2202 (92.37)	182 (7.63)		0.86–1.45	1020 (85.57)	162 (13.59)	10 (0.84)	
rs4252707		G	*A*			GG	GA	AA	
Case	0.239	671 (59.7)	453 (40.3)	***0.000101***	1.34	207 (36.83)	257 (45.73)	98 (17.44)	***0.000157***
Control	0.418	1584 (66.44)	800 (33.56)	***0.002424***	1.16–1.55	520 (43.62)	544 (45.64)	128 (10.74)	***0.003768***

CI: confidence interval; OR: odds ratio.

Risk allele were in italic, and significant *P* values were in italic bold.

Corrected *P* values were underlined after Bonferroni correction (*P* × 24).

OR referred to the risk allele odds ratio in cases and controls, and the reference allele for OR calculation was non-risk allele.

*Obtained in logistic regression models with adjustment for age and gender.

**Table 3:** Stratification analyses for association between rs4252707 genotypes and glioma risk.

rs4252707	Genotype Counts (Case/Control)			*P*-value^a^	H-WE *P*-value (Case/Control)	OR^a^ (95% CI)
	GG	GA	AA			
Age (years)						
<18 Children	28/76	38/83	17/19	0.0783	0.536/0.602	1.46 (0.85–2.52)
≥18 Adults	179/444	219/461	81/109	***0.0016***	0.318/0.510	1.31 (1.12–1.54)
IR exposure history						
No	205/516	247/532	85/118	***0.0013***	0.171/0.646	1.29 (1.11–1.50)
Yes	2/4	10/12	13/10	0.3947	0.968/0.899	1.61 (0.70–3.70)
Smoking status						
Smoker	101/252	122/260	43/51	***0.0068***	0.547/0.165	1.36 (1.09–1.68)
Non-smoker	106/268	135/284	55/77	***0.0186***	0.303/0.895	1.32 (1.08–1.62)
Histology of non-GBM						
Astrocytomas	107/520	133/544	50/128	***0.0048***	0.434/0.418	1.33 (1.10–1.60)
Other gliomas*	100/520	124/544	48/128	***0.0156***	0.376/0.418	1.34 (1.11–1.63)
Oligodendrogliomas	43/520	53/544	23/128	***0.0153***	0.363/0.418	1.43 (1.08–1.85)
Enpendymomas	37/520	45/544	20/128	***0.0215***	0.351/0.418	1.41 (1.06–1.89)
Mixed glioma	20/520	26/544	5/128	0.7159	0.407/0.418	1.08 (0.71–1.64)

*Other gliomas including oligodendrogliomas, enpendymomas or mixed glioma.

CI: confidence interval; OR: odds ratio.

*P* values (p < 0.05) are in italic bold to indicate significant association signals.

OR referred to the AA + GA genotypes, and the reference genotype for OR calculation was the GG genotype.

^a^Obtained in logistic regression models with adjusted for age and gender (GG vs GA vs AA).

**Table 4:** False-positive report probability results for significant findings in subgroups.

rs4252707	P-value^a^	OR^a^	Statistical power^b^	Prior probability				
				0.25	0.1	0.01	0.001	0.0001
≥18 Adults	0.0016	1.31	0.857	***0.0056***	***0.0165***	***0.1560***	0.6510	0.9492
No IR exposure	0.0013	1.29	0.862	***0.0045***	***0.0134***	***0.1299***	0.6011	0.9378
Smoker	0.0068	1.36	0.732	***0.0271***	***0.0772***	0.4791	0.9027	0.9893
Non-smoker	0.0186	1.32	0.689	***0.0749***	***0.1955***	0.7277	0.9642	0.9963
Astrocytomas	0.0048	1.33	0.765	***0.0185***	***0.0535***	0.3832	0.8624	0.9843
Other gliomas*	0.0156	1.34	0.761	***0.0579***	***0.1558***	0.6699	0.9534	0.9951
oligodendrogliomas	0.0153	1.43	0.613	***0.0697***	***0.1834***	0.7119	0.9614	0.9960
enpendymomas	0.0215	1.41	0.513	***0.1117***	0.2739	0.8058	0.9767	0.9976

^*^Other gliomas including oligodendrogliomas, enpendymomas or mixed glioma.

OR, odds ratio.

^a^P values and OR were from Table 3.

^b^Statistical power was calculated using the number of observations in the subgroup and the OR and P values in this table.

**Figure 1:**
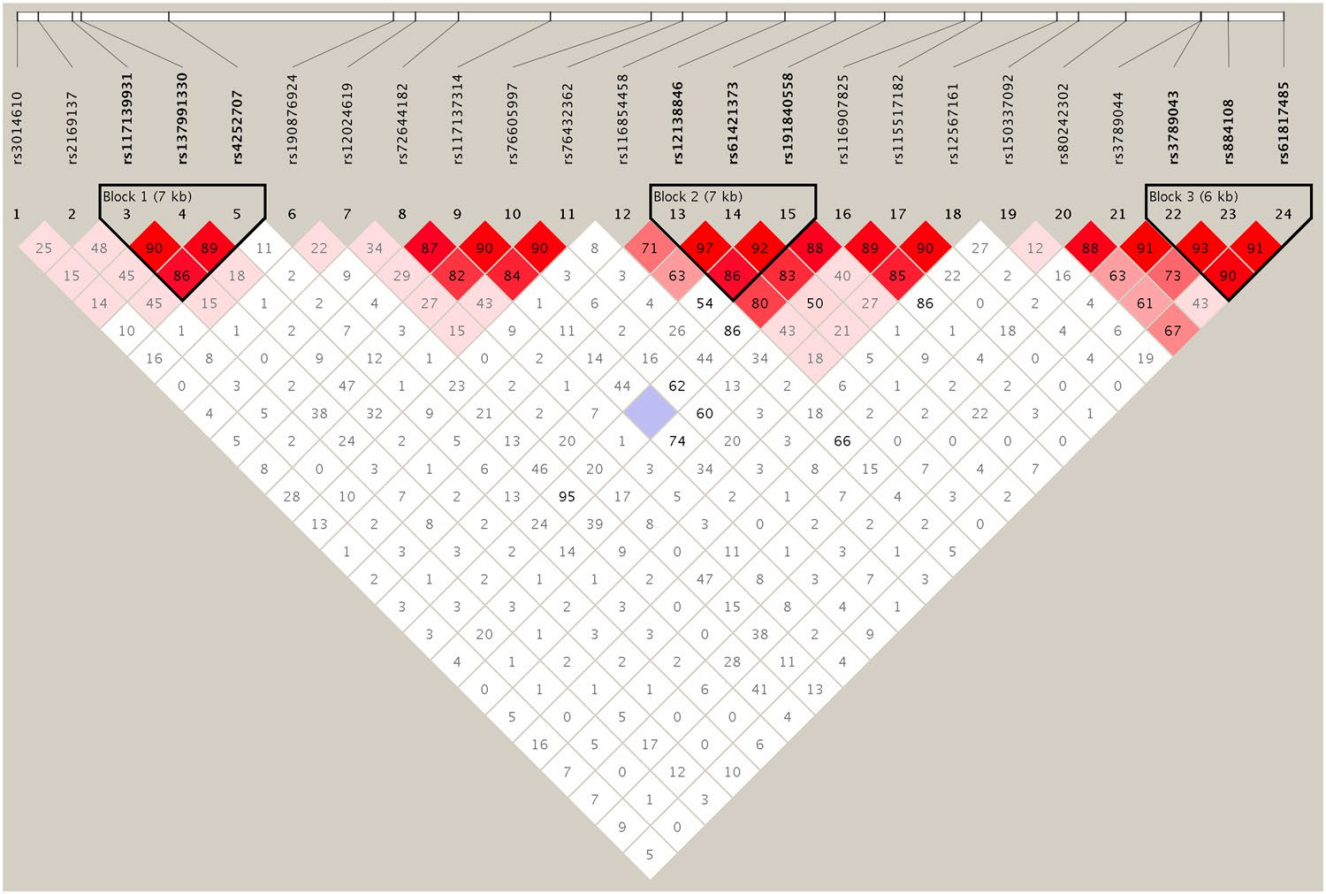
LD structure based on genotype datasets. The LD blocks are indicated as shaded boxes.

Based on the genotype data from this study, we constructed the linkage disequilibrium (LD) structure of all genotyped SNPs. As shown in Fig. 1, three LD blocks were identified in the data, and the SNP rs4252707 was included in block 1. Haplotypic association analyses were performed to test the LD block including rs4252707. The correlations between haplotypic frequencies and glioma risk are presented in Table 5, and a significant global *P* value (global *P* = 0.015) was secured from the LD region (Table 5). These results provided additional evidence for the significant correlation of the SNP of rs4252707 with the risk of non-GBM susceptibility. Haplotype of TGA was positively correlated with the risk of non-GBM glioma. Given its more prevalence in controls, the TGG haplotype may provide a protective effect (Table 5).

**Table 5:** Haplotype frequencies and association analyses.

Haplotype	Haplotype Frequencies (%)			Global *P-*value^b^
	Case	Control	*P*-value^a^	
Block 1, rs117139931- rs137991330-rs4252707				
*TGG*	58.50	65.64	***0.00004***	***0.015***
*TGA*	32.23	26.09	***0.00016***	
CAA	7.00	6.89	0.90346	

Significant P values were in italic bold.

Rare haplotypes were not shown, if the frequency less than 1%.

^a^Based on 100000 permutations.

^b^Based on comparison of frequency distribution of all haplotypes for the combination of SNPs.

## Discussion

Candidate gene-based association studies have successfully mapped susceptibility for many complex diseases^22–28^. Accumulating evidence indicates that many genes contribute to glioma susceptibility and risk, but the underlying molecular mechanisms remain unknown. Several studies have reported that *MDM4* is involved in GBM^29,30^, but to date, only one study has investigated the association between *MDM4* polymorphisms and non-GBM glioma occurrence in patients of European heritage^18^. Here, we conducted a case-control study of the *MDM4* gene to evaluate the potential associations between genetic variations and glioma risk in Han Chinese population. To the best of our knowledge, this is the first study focusing on the relationship between *MDM4* and glioma risk in Asians. Our study showed that rs4252707 SNP was strongly correlated with the non-GBM risk. Patients with this disease showed significantly higher frequencies of the A allele (*P* = 0.00010) and the AA genotype (*P* = 0.00016) than normal controls, which is consistent with what was observed in the European population^18^. Moreover, the ORs of rs4252707 in the study were similar with those reported by Melin *et al*.^18^. Considering the limitations to drawing a conclusion from the analysis of SNPs^31–35^, we examined LD structure using genotype data based on candidate SNPs to further strengthen our findings. The results revealed that the haplotype T-G-G (rs117139931 - rs137991330 - rs4252707) containing rs4252707 was also strongly correalted with the increased non-GBM risk (*P* = *0.00016*). These findings suggest that the rs4252707 SNP of the *MDM4* gene may affect the susceptibility of non-GBM glioma in Han Chinese individuals.

It has been shown that the p53 pathway is inactivated in almost all human cancers^36^. Approximately half of human malignancies have been estimated to carry mutations in the TP53 gene itself, whereas the remaining tumors with wild type TP53 contain genetic alterations in other key regulatory genes in the p53 pathway^37,38^. MDM4 is one of the key negative regulators of p53, and its overexpression or amplification contributes to carcinogenesis by inhibiting p53 tumor suppressor activity^39^. Genetic amplification of the *MDM2* or *MDM4* genes, among others, can result in aberrant protein expression and suppression of the p53 response over the course of tumor development^40,41^. More importantly, previous studies suggest that polymorphisms of the *MDM2* or *MDM4* genes may contribute to increased basal expression of these important p53 antagonists and thereby increase cancer susceptibility^42,43^. For example, the SNP rs4245739 in *MDM4* is significantly associated with ovarian cancer, retinoblastoma and breast cancer^13,44,45^. Allele C of rs4245739 can cause decreased expression of *MDM4* mRNA^46^, leading to a protective effect against cancer. In our study, the associated SNP rs4252707, which is located in intron 8 of the *MDM4* gene and in LD with the *MDM4* promoter, may enhance *MDM4* expression and ultimately promote tumorigenesis. Therefore, based on our results, we hypothesize that the SNP rs4252707 may contribute to the risk of developing non-GBM glioma by somehow influencing the expression of *MDM4*, such as by affecting the splice sites or by being closely linked with some functional variations. However, the potential biological explanations for the significant associations should be confirmed by direct biological evidence in future research.

The interesting finding of our study was that the increase in the risk of developing non-GBM glioma associated with the AA genotype of rs4252707 remained significant for subjects without a history of IR exposure but not for subjects with a history of IR exposure. Thus far, the only established environmental risk factor for non-GBM glioma is exposure to therapeutic or high-dose IR^4,47^. A previous study found that genetic variability in DNA repair genes contributes to a hypersensitivity to IR and increased susceptibility to cancer^48^. Here, the association of rs4252707 was not significant in the cohort reporting IR exposure, but the result should be interpreted by cautions. Because the subset of the study subjects was very small, there was no sufficient power to test the association in the subgroup. Moreover, given of the lack of biological experimental evidence, it is still unknown whether this SNP may cause cancer through an effect on DNA damage and repair pathways or by changing the expression and activity of the p53 pathway. In the present study, only a small fraction of samples had the history of IR exposure. A possible explanation is that subjects may not be aware of exposed low-dose IR or IR of unknown sources. Additionally, we further analyzed the association between rs4252707 genotypes and environmental factors, such as age, tumor type and smoking history, that may influence the occurrence of glioma. We found that the rs4252707 genotype is closely associated with the occurrence of non-GBM glioma in the adult subgroup but is not related to smoking or the type of glioma, which is consistent with a previous epidemiological study suggesting that glioma is usually more prevalence in adults than in children^49^.

Although similar results were obtained in two distinctly different ethnic groups (Han Chinese and Europeans), our study has some limitations that need to be addressed. First, we only evaluated common SNPs across the *MDM4* gene. Given the lack of rare variant data, these SNPs may not be sufficient to comprehensively evaluate glioma risk associated with the *MDM4* gene. Second, further study is needed to determine whether the AA genotype of rs4252707 affects the expression of *MDM4*. Third, increasing evidences suggest an association between the history of allergy or asthma and glioma risk^50,51^. However, because of the lack of the relevant environmental data in our cohort, it is unclear whether this would affect the observed association in the study. The revised 2016 WHO classification redefines low grade gliomas with morphological and molecular subtype information. As known, low grade gliomas are histologically and genetically heterogeneous. Although histological grade is still effective for the diagnosis of glioma, the prognosis of patients are more closely correlated with molecular subtype than with histological grade. However, there were some difficulties in understanding genetic heterogeneity of lower grade glioma, as we have not yet obtained the molecular subtype information of the patients of glioma. Thus, future studies would be desired to confirm our results. Finally, this study was performed at a single center, which may potentially limit the generalizability of the findings. Therefore, a larger and well-designed analysis based on different populations is required to fully elucidate the relevant mechanisms in future research.

In summary, our study provides strong evidence that the *MDM4* gene is tightly associated with genetic susceptibility risk of non-GBM in Han Chinese individuals. This significant finding is further confirmed by haplotypic analysis. More studies focusing on the pathological mechanisms of non-GBM glioma and *MDM4* are still needed to unravel the role of *MDM4* in the onset and development of non-GBM glioma.

## Electronic supplementary material

Supplemental materials
