# Supplementary material for: MDM4 contributes to the increased risk of glioma susceptibility in Han Chinese population
Source: Sci Rep. 2018 Jul 23;8:11093. doi: 10.1038/s41598-018-29468-6 (PMC6056491; doi:10.1038/s41598-018-29468-6)
Supplement: Supplementary file 1 — Supplemental materials [file 41598_2018_29468_MOESM1_ESM.docx]

***Title***: *MDM4* contributes to the increased risk of glioma susceptibility in Han Chinese population

***Author names and affiliations***: Peng Sun ^1,3^, Feng Yan ^2^, Wei Fang ^3^, Junjie Zhao ^1^, Hu Chen ^3^ , Xudong Ma ^1^ and Jinning Song ^1^

^1^ Department of Neurosurgery, the First Affiliated Hospital of Xi’an Jiaotong University, Xi'an, Shaanxi, China;

^2^ Department of Clinical Nutrition, Xijing Hospital, the Fourth Military Medical University, Xi'an, Shaanxi, China;

^3^ Department of Neurosurgery, Tangdu Hospital, the Fourth Military Medical University, Xi'an, Shaanxi, China.

***Corresponding Author***:

Jinning Song, M.D. & Ph.D., Department of Neurosurgery, the First Affiliated Hospital of Xi’an Jiaotong University, No.277, Yanta West Road, Yanta District, Xi'an, Shaanxi, China, 710061.

Tel: 86-29- 85323983; Fax: 86-29- 85323983; E-mail: jnsongneu@163.com

Table S1 The information of 24 genotyped tag SNPs of *MDM4*

| SNP | Chromosome | Position | Base change |
| --- | --- | --- | --- |
| rs3014610 | 1 | 204527143 | A/T |
| rs2169137 | 1 | 204528785 | C/G |
| rs117139931 | 1 | 204531485 | T/C |
| rs137991330 | 1 | 204532159 | G/A |
| rs4252707 | 1 | 204539019 | G/A |
| rs190876924 | 1 | 204556662 | A/C |
| rs12024619 | 1 | 204558388 | G/A |
| rs72644182 | 1 | 204561735 | C/T |
| rs117137314 | 1 | 204568980 | A/G |
| rs76605997 | 1 | 204576856 | G/T |
| rs76432362 | 1 | 204579351 | C/T |
| rs116854458 | 1 | 204582792 | G/A |
| rs12138846 | 1 | 204587350 | A/T |
| rs61421373 | 1 | 204591312 | C/T |
| rs191840558 | 1 | 204595177 | C/T |
| rs116907825 | 1 | 204601448 | A/T |
| rs115517182 | 1 | 204602790 | C/T |
| rs12567161 | 1 | 204608704 | G/A |
| rs150337092 | 1 | 204610398 | T/A |
| rs80242302 | 1 | 204614094 | T/A |
| rs3789044 | 1 | 204619973 | G/A |
| rs3789043 | 1 | 204620022 | C/T |
| rs884108 | 1 | 204622109 | G/A |
| rs61817485 | 1 | 204626464 | A/G |

Table S2 Allele and genotype frequencies of single SNP association analyses for other 21 SNPs

| SNP ID | Allele Freq (%) | | *P-*value | Genotype Freq (%) | | | *P-*value | HWE *P*-value |
| --- | --- | --- | --- | --- | --- | --- | --- | --- |
| rs3014610 | A | T |  | AA | AT | TT |  |  |
| CASE | 942(83.81) | 182(16.19) | 0.726646 | 394(70.11) | 154(27.4) | 14(2.49) | 0.940166 | 0.819 |
| CONTROL | 2009(84.27) | 375(15.73) |  | 845(70.89) | 319(26.76) | 28(2.35) |  | 0.744 |
| rs2169137 | C | G |  | CC | CG | GG |  |  |
| CASE | 1028(91.46) | 96(8.54) | 0.701096 | 470(83.63) | 88(15.66) | 4(0.71) | 0.706751 | 0.957 |
| CONTROL | 2171(91.07) | 213(8.93) |  | 988(82.89) | 195(16.36) | 9(0.76) |  | 0.854 |
| rs190876924 | A | C |  | AA | AC | CC |  |  |
| CASE | 1063(94.57) | 61(5.43) | 0.381804 | 504(89.68) | 55(9.79) | 3(0.53) | 0.400306 | 0.269 |
| CONTROL | 2271(95.26) | 113(4.74) |  | 1084(90.94) | 103(8.64) | 5(0.42) |  | 0.136 |
| rs12024619 | G | A |  | GG | GA | AA |  |  |
| CASE | 1049(93.33) | 75(6.67) | 0.929004 | 491(87.37) | 67(11.92) | 4(0.71) | 0.608825 | 0.310 |
| CONTROL | 2223(93.25) | 161(6.75) |  | 1035(86.83) | 153(12.84) | 4(0.34) |  | 0.509 |
| rs72644182 | C | T |  | CC | CT | TT |  |  |
| CASE | 918(81.67) | 206(18.33) | 0.651762 | 377(67.08) | 164(29.18) | 21(3.74) | 0.785069 | 0.550 |
| CONTROL | 1962(82.3) | 422(17.7) |  | 807(67.7) | 348(29.19) | 37(3.1) |  | 0.945 |
| rs117137314 | A | G |  | AA | AG | GG |  |  |
| CASE | 1075(95.64) | 49(4.36) | 0.481411 | 515(91.64) | 45(8.01) | 2(0.36) | 0.512407 | 0.346 |
| CONTROL | 2292(96.14) | 92(3.86) |  | 1103(92.53) | 86(7.21) | 3(0.25) |  | 0.339 |
| rs76605997 | G | T |  | GG | GT | TT |  |  |
| CASE | 1073(95.46) | 51(4.54) | 0.683060 | 513(91.28) | 47(8.36) | 2(0.36) | 0.680921 | 0.412 |
| CONTROL | 2283(95.76) | 101(4.24) |  | 1095(91.86) | 93(7.8) | 4(0.34) |  | 0.184 |
| rs76432362 | C | T |  | CC | CT | TT |  |  |
| CASE | 1033(91.9) | 91(8.1) | 0.730157 | 477(84.88) | 79(14.06) | 6(1.07) | 0.959064 | 0.189 |
| CONTROL | 2199(92.24) | 185(7.76) |  | 1016(85.23) | 167(14.01) | 9(0.76) |  | 0.461 |
| rs116854458 | G | A |  | GG | GA | AA |  |  |
| CASE | 1081(96.17) | 43(3.83) | 0.521843 | 519(92.35) | 43(7.65) | 0(0) | 0.514030 | 0.346 |
| CONTROL | 2303(96.6) | 81(3.4) |  | 1111(93.2) | 81(6.8) | 0(0) |  | 0.225 |
| rs12138846 | A | T |  | AA | AT | TT |  |  |
| CASE | 756(67.26) | 368(32.74) | 0.813550 | 255(45.37) | 246(43.77) | 61(10.85) | 0.971306 | 0.885 |
| CONTROL | 1613(67.66) | 771(32.34) |  | 548(45.97) | 517(43.37) | 127(10.65) |  | 0.758 |
| rs61421373 | C | T |  | CC | CT | TT |  |  |
| CASE | 707(62.9) | 417(37.1) | 0.875331 | 224(39.86) | 259(46.09) | 79(14.06) | 0.985394 | 0.766 |
| CONTROL | 1493(62.63) | 891(37.37) |  | 470(39.43) | 553(46.39) | 169(14.18) |  | 0.757 |
| rs191840558 | C | T |  | CC | CT | TT |  |  |
| CASE | 1013(90.12) | 111(9.88) | 0.528284 | 457(81.32) | 99(17.62) | 6(1.07) | 0.817344 | 0.806 |
| CONTROL | 2132(89.43) | 252(10.57) |  | 954(80.03) | 224(18.79) | 14(1.17) |  | 0.835 |
| rs116907825 | A | T |  | AA | AT | TT |  |  |
| CASE | 1075(95.64) | 49(4.36) | 0.481411 | 515(91.64) | 45(8.01) | 2(0.36) | 0.512407 | 0.346 |
| CONTROL | 2292(96.14) | 92(3.86) |  | 1103(92.53) | 86(7.21) | 3(0.25) |  | 0.339 |
| rs115517182 | C | T |  | CC | CT | TT |  |  |
| CASE | 1033(91.9) | 91(8.1) | 0.730157 | 477(84.88) | 79(14.06) | 6(1.07) | 0.959064 | 0.189 |
| CONTROL | 2199(92.24) | 185(7.76) |  | 1016(85.23) | 167(14.01) | 9(0.76) |  | 0.461 |
| rs12567161 | G | A |  | GG | GA | AA |  |  |
| CASE | 880(78.29) | 244(21.71) | 0.744560 | 345(61.39) | 190(33.81) | 27(4.8) | 0.948358 | 0.898 |
| CONTROL | 1878(78.78) | 506(21.22) |  | 741(62.16) | 396(33.22) | 55(4.61) |  | 0.822 |
| rs150337092 | T | A |  | TT | TA | AA |  |  |
| CASE | 1081(96.17) | 43(3.83) | 0.521843 | 519(92.35) | 43(7.65) | 0(0) | 0.514030 | 0.346 |
| CONTROL | 2303(96.6) | 81(3.4) |  | 1111(93.2) | 81(6.8) | 0(0) |  | 0.225 |
| rs80242302 | T | A |  | TT | TA | AA |  |  |
| CASE | 1032(91.81) | 92(8.19) | 0.727539 | 475(84.52) | 82(14.59) | 5(0.89) | 0.809182 | 0.488 |
| CONTROL | 2197(92.16) | 187(7.84) |  | 1014(85.07) | 169(14.18) | 9(0.76) |  | 0.504 |
| rs3789044 | G | A |  | GG | GA | AA |  |  |
| CASE | 753(66.99) | 371(33.01) | 0.784670 | 251(44.66) | 251(44.66) | 60(10.68) | 0.952157 | 0.815 |
| CONTROL | 1586(66.53) | 798(33.47) |  | 527(44.21) | 532(44.63) | 133(11.16) |  | 0.942 |
| rs3789043 | C | T |  | CC | CT | TT |  |  |
| CASE | 1013(90.12) | 111(9.88) | 0.740485 | 458(81.49) | 97(17.26) | 7(1.25) | 0.894430 | 0.472 |
| CONTROL | 2157(90.48) | 227(9.52) |  | 977(81.96) | 203(17.03) | 12(1.01) |  | 0.688 |
| rs884108 | G | A |  | GG | GA | AA |  |  |
| CASE | 987(87.81) | 137(12.19) | 0.614364 | 433(77.05) | 121(21.53) | 8(1.42) | 0.866839 | 0.891 |
| CONTROL | 2079(87.21) | 305(12.79) |  | 907(76.09) | 265(22.23) | 20(1.68) |  | 0.899 |
| rs61817485 | A | G |  | AA | AG | GG |  |  |
| CASE | 627(55.78) | 497(44.22) | 0.723600 | 174(30.96) | 279(49.64) | 109(19.4) | 0.938681 | 0.880 |
| CONTROL | 1345(56.42) | 1039(43.58) |  | 378(31.71) | 589(49.41) | 225(18.88) |  | 0.868 |
